# Relapse of acute myocarditis associated with *Campylobacter jejuni* enterocolitis

**DOI:** 10.1002/ccr3.3235

**Published:** 2020-08-12

**Authors:** Shizuka Yaita, Masaki Tago, Yoshio Hisata, Motoshi Fujiwara, Shu‐ichi Yamashita

**Affiliations:** ^1^ Department of General Medicine Saga University Hospital Saga Japan

**Keywords:** acute myocarditis, *Campylobacter jejuni*, relapse

## Abstract

Chest pain in a patient with *Campylobacter jejuni* infection may be caused by acute myocarditis associated with *C jejuni* infection. Because the myocarditis associated with *C jejuni* infection can recur, careful follow‐up is required even after the improvement of chest pain and electrocardiography abnormalities.

## Graphical abstract

As the myocarditis associated with *Campylobacter jejuni* infection can recur, careful follow‐up is required even after the improvement of chest pain and ECG abnormalities.

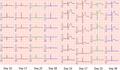

## 1 INTRODUCTION

*Campylobacter* infection causes enterocolitis and may also cause various complications such as Guillain‐Barré syndrome, reactive arthritis, inflammatory bowel disease, arteritis with aortic dissection, cholecystitis, and acute myocarditis.^1^ Acute myocarditis associated with *Campylobacter jejuni* infection is common in males aged 10‐49 years. The affected patients present 3‐5 days after the onset of gastroenteritis with chest tightness, chest pain, dyspnea, abnormalities on electrocardiography (ECG), and elevated levels of serum myocardial enzymes.^2^ The diagnosis of acute myocarditis associated with *C jejuni* infection is based on the detection of *C jejuni* in stool culture or a positive serum antibody titer.^1^ Although the prognosis is usually good, there are some reports of mortality or residual mitral valve prolapse.^2^, ^3^ There has been no report of relapse of myocarditis associated with *C jejuni* during follow‐up of several weeks.^2^, ^4^, ^5^, ^6^ We herein report the first case of acute relapse of myocarditis associated with *C jejuni*.

## 2 CASE HISTORY/EXAMINATION

A 16‐year‐old Japanese male developed nonhemorrhagic watery diarrhea, headache, and abdominal pain 4 days prior to hospital admission. He had no family history of diarrhea, dietary abnormalities, travel, or contact with animals. He did not have any relevant medical history (including Kawasaki disease) other than bronchial asthma. Two days before hospital admission, a primary care doctor had prescribed ciprofloxacin 400 mg/d and loperamide 2 mg/d. As the patient's symptoms were worsening, he was admitted with a diagnosis of acute enteritis and dehydration (Day 1). On the night of Day 1, the patient developed persistent precordial pain at rest that was exacerbated by deep inspiration; this pain persisted until the evening of Day 2.

At the onset of precordial pain, the patient was alert, with a temperature of 39.1°C, blood pressure of 104/56 mm Hg, regular pulse rate of 96 beats per minute, and oxygen saturation of 97% on room air without dyspnea. Physical examination revealed right lower abdominal tenderness without guarding, but no abnormal cardiac or respiratory sounds such as arrhythmia and pericardial friction rub.

**FIGURE 1:**
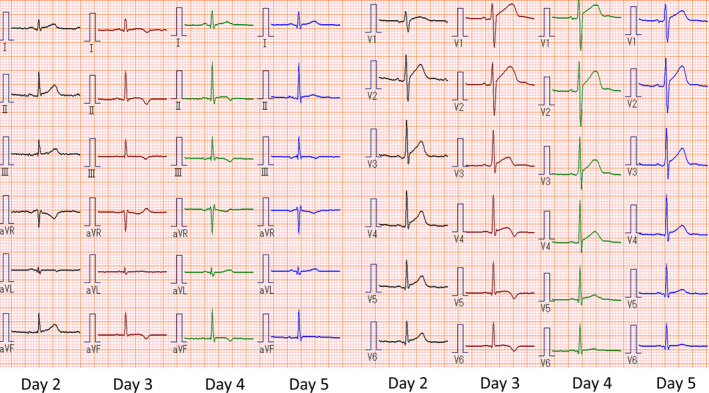
Electrocardiographic changes at the onset of the initial symptoms. There are ST elevations in leads II, aVf, V3, V4, V5, and V6 on Day 2, T wave inversions on days 3 and 4, and normal patterns on Day 5

Laboratory examination results on Day 2 showed a white blood cell count of 10 300/L (neutrophils 76.8%, lymphocytes 8%, monocytes 8.1%, eosinophils 0%, basocytes 0.4%), C‐reactive protein level of 12.7 mg/dL, aspartate aminotransferase level of 167 IU/dL, alanine aminotransferase level of 78 IU/dL, lactase dehydrogenase level of 414 IU/dL, creatine kinase level of 1808 IU/dL, troponin T level of 1.71 ng/mL, and N‐terminal probrain natriuretic peptide level of 404.8 pg/mL. A stool culture performed on Day 6 showed the presence of *C jejuni* and was negative for blood. ECG showed ST elevation in leads II, aVf, V3, V4, V5, and V6 (Figure 1). Chest radiography was normal, and transthoracic echocardiography (TTE) did not show asynergy of ventricular movement or pericardial effusion. Paired sera testing showed no significant changes in the titers of antiviral antibodies for any major viruses reported to cause viral myocarditis, including adenovirus, Epstein‐Barr virus, cytomegalovirus, echovirus, coxsackievirus, or parvovirus.

## 3 DIFFERENTIAL DIAGNOSES, INVESTIGATIONS, AND TREATMENT

Myocardial disease was suspected because of the precordial pain, ST elevations on ECG, and myocardial enzyme elevations. Acute coronary syndrome was considered unlikely because of the patient's young age, no history of Kawasaki disease, and normal TTE findings; therefore, he was diagnosed with acute myocarditis. Coronary angiography was not performed because of the low risk of coronary artery disease and the patient's history of bronchial asthma. The diagnosis of acute relapse of myocarditis associated with *C jejuni* enterocolitis was based on the negative blood culture results, changes in paired viral titers, positive stool culture result for *C jejuni,* relapse after the resolution of chest pain, laboratory data, and ECG findings.

As the patient was hemodynamically stable, he was initially treated conservatively while awaiting the stool culture results. Antibiotics were not administered, as the chest pain and elevation of myocardial enzymes had improved by the time the positive stool culture for *C jejuni* was revealed on Day 6.

## 4 OUTCOME AND FOLLOW‐UP

**FIGURE 2:**
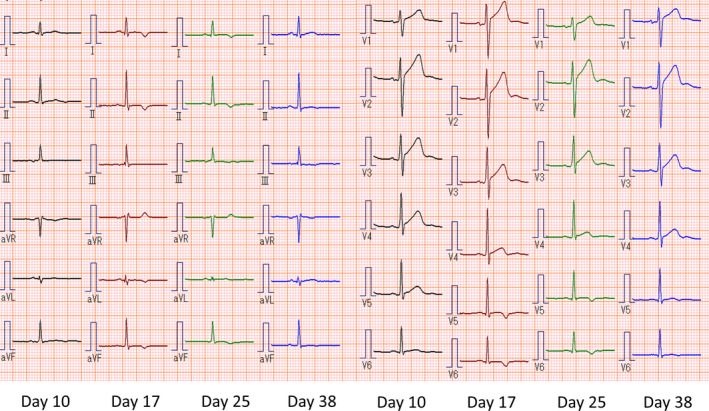
Electrocardiographic changes at the time of relapse of myocarditis. Although follow‐up electrocardiography on Day 10 shows no abnormalities, electrocardiography performed on Day 17 reveals new inverted T waves in a wide range of leads that remain until Day 25, and then normalize on Day 38

The chest pain disappeared on Day 2. The elevated levels of creatine kinase, creatine kinase‐muscle/brain, and C‐reactive protein on Day 1 had decreased to normal levels by Day 6 (Table 1). Serial ECG examinations showed ST elevation on Day 2, inversion of T waves on days 3 and 4, and no abnormalities on Day 5 (Figure 1). As the patient's symptoms and data had improved, he was discharged on Day 6. No abnormalities were found on follow‐up ECG performed on Day 10 (Figure 2). The patient revisited our hospital on Day 17 because of crampy precordial pain that had lasted for a few minutes at night. The chest pain had already disappeared on arrival, and there were no abnormal findings on blood tests (Table 1) or TTE. However, ECG performed on Day 17 revealed inverted T waves in multiple leads (Figure 2), which led to the diagnosis of acute relapse of myocarditis. As the patient had stable hemodynamics, he was carefully monitored as an outpatient. The ECG abnormalities remained until Day 25 and then normalized on Day 38 (Figure 2). The patient has had no subsequent symptoms of relapse.

**TABLE 1:** Results of blood testing performed at the onset of myocarditis and at the time of relapse

	At the onset			At relapse	
	Day 2	Day 3	Day 6	Day 17	Day 38
WBC (/μL)	10 300	8 100	10 100	6 700	8 000
Seg (%)	76.8	71.2	65.3	53.2	50.6
Lymph (%)	12.4	15.5	23.0	34.5	36.2
Mono (%)	10.2	11.8	7.2	9.1	6.8
Eosino (%)	0.3	1.1	3.6	2.2	5.4
Baso (%)	0.3	0.4	0.9	1.0	1.0
AST (IU/L)	167	73	29	29	20
LDH (IU/L)	414	387	264	159	147
CK (IU/L)	1 808	564	49	53	71
CK‐MB (IU/L)	123	17	6	5	5
CRP (mg/dL)	12.68	8.23	2.60	0.29	0.06
Troponin T (ng/mL)	1.76			0.007	0.008
NT‐proBNP (IU/L)	404.8				33.8

Abbreviations: Baso, basocytes; CK‐MB, creatine kinase‐muscle/brain; CPK, creatine phosphokinase; CRP, C‐reactive protein; Eosino, eosinophils; Lymph, lymphocytes; Mono, monocytes; NT‐proBNP, N‐terminal probrain natriuretic peptide; Seg, segmented neutrophils; WBC, white blood cell count.

## 5 DISCUSSION

This is the first case report of acute relapse of myocarditis associated with *C jejuni* enterocolitis. *Campylobacter* infection is a well‐known cause of enterocolitis that can be complicated with acute myocarditis.^1^, ^2^ In most cases, acute myocarditis associated with *C jejuni* develops approximately 1 week after the onset of enterocolitis.^2^, ^3^, ^4^, ^6^, ^7^, ^8^, ^9^ Although most patients have a good prognosis, the early detection of *C jejuni*, early diagnosis of acute myocarditis, and early commencement of treatment are essential, as some cases are fatal or have residual permanent cardiac damage.^3^ When patients present with chest pain or chest tightness with a recent history of enterocolitis, physicians must consider acute myocarditis as a differential diagnosis.^1^, ^2^, ^4^

The pathogenesis of myocarditis is usually classified as infectious, toxic, or immunological; however, the underlying mechanism of acute myocarditis associated with *C jejuni* infection remains unclarified.^1^, ^10^ There has been only one autopsy case report of a patient with acute myocarditis associated with *C jejuni* infection.^3^ This previous autopsy case revealed myocardial inflammation mainly comprising neutrophil infiltration, suggesting that the mechanism of myocardial injury was either direct infection of *C jejuni* in the myocardium or damage due to the toxin produced by *C jejuni*.^3^ Further study of accumulated cases of relapse of myocarditis is required to clarify the mechanism of relapse that occurred in the present case, and to determine the pathological mechanism of myocardial inflammation associated with *C jejuni* infection.

A diagnosis of acute myocarditis associated with *C jejuni* infection requires the detection of *C jejuni* (usually by stool culture or serum antibody measurement) plus the identification of myocarditis.^5^ Although the Dallas criteria state that a definitive diagnosis of myocarditis requires an endocardial myocardial biopsy (EMB), a joint statement from the American Heart Association, American College of Cardiology Foundation, and European Society of Cardiology only advocates the use of EMB when the benefit of performing the biopsy outweighs the risk of complications.^11^ Therefore, it is not appropriate to use EMB to diagnose acute myocarditis associated with *C jejuni* infection, as this condition has a relatively good prognosis. A literature search did not identify any case report in which EMB was performed to diagnose acute myocarditis associated with *C jejuni* infection; several cases were diagnosed based on the combination of clinical findings such as chest pain, laboratory findings including elevated levels of creatine kinase, creatine kinase‐muscle/brain, and troponin T, and ST changes on ECG.^2^, ^3^, ^4^, ^8^, ^9^, ^12^ In the current case, we considered it inappropriate to perform EBM owing to the patient's excellent general condition. Although cardiac magnetic resonance imaging has recently been used in the diagnosis or evaluation of myocarditis, including acute myocarditis associated with *C jejuni* infection,^5^, ^6^, ^9^, ^13^, ^14^, ^15^ this method was not performed in the present case.

Acute myocarditis associated with *C jejuni* infection is usually treated with antibiotics, especially ciprofloxacin or macrolide antibiotics, as well as conservative symptomatic treatment. However, there is no available evidence regarding the appropriate dosage, treatment duration, or pharmacological efficacy.^2^, ^4^, ^5^, ^7^, ^9^, ^12^ Furthermore, the emergence of quinolone‐resistant strains of *C jejuni* has become a global problem.^1^ Acute myocarditis associated with *C jejuni* infection is also treated with medications that are routinely used to treat heart failure, such as diuretics, angiotensin‐converting enzyme inhibitors, angiotensin receptor blockers, or β‐blockers, with the aim of protecting the myocardium and providing symptomatic treatment, just as in other forms of myocarditis caused by more common pathogens such as viruses.^4^ Although there have been a few case reports of severe acute myocarditis associated with *C jejuni* infection,^2^, ^3^ the present patient showed relatively quick improvements in chest pain and ECG abnormalities, both in the first episode and the relapse, which enabled us to treat him conservatively with watchful waiting.

When a patient presents with chest pain associated with *C jejuni* enterocolitis, it is essential to consider a diagnosis of acute myocarditis. Furthermore, careful follow‐up is required owing to the rare but possible relapse of myocarditis.

## CONSENT STATEMENT

Published with written consent of the patient.

## CONFLICT OF INTEREST

None declared.

## AUTHORS' CONTRIBUTIONS

SY and MT: involved in literature search, concept, and drafting; YH: involved in literature search, concept, and clinical care of the patient; MF: involved in concept, drafting of the manuscript, and clinical care of the patient; SY: involved in concept and revision of the article.

## ETHICAL APPROVAL

The patient gave permission for the publication of this case report. This manuscript conforms to the provisions of the Declaration of Helsinki in 1995 (as revised in Brazil 2013).
